# Impact of Gastric Sleeve Surgery on Plasma Retinol Binding Protein 4 and Adipsin Levels in Healthy Male Population

**DOI:** 10.12669/pjms.36.7.2329

**Published:** 2020

**Authors:** Suad Alshubrami, Khalid Al-Regaiey, Assim A. Alfadda, Muhammad Iqbal

**Affiliations:** 1 Suad Alshubrami Department of Physiology, College of Medicine, King Saud University, Riyadh, Saudi Arabia. Current Address: Director of Academic and Training Affairs Continuous Professional, King Salman Specialist Hospital, Hail, Saudi Arabia; 2 Khalid Al-Regaiey Department of Physiology, College of Medicine, King Saud University, Riyadh, Saudi Arabia; 3 Assim A. Alfadda Obesity Research Center, Department of Medicine, College of Medicine, King Saud University, Riyadh, Saudi Arabia; 4 Muhammad Iqbal Department of Physiology,

**Keywords:** Sleeve gastrectomy, Glycaemic indicators, RBP4, Adipsin

## Abstract

**Objectives:**

Bariatric surgery provides most substantial and sustainable weight loss measures in individuals with obesity. Caloric restriction is not only intervention, changes in hormonal secretions are also leading contributory mechanisms to reduce body weight and improve the glycaemic control. The aim of this study was to evaluate the impact of gastric sleeve surgery on plasma retinol binding protein 4 (RBP4) and adipsin levels among Saudi male obese population.

**Methods:**

This prospective study was conducted in the Departments of Physiology and Surgery, College of Medicine, King Saud University. Thirty-three obese (BMI>38.3) male patients age ranged from 25 to 50 years were recruited. RBP4 and adipsin levels were analyzed before and 6-12 months after gastric sleeve surgery by ELISA along with plasma glucose, insulin, HOMA-IR and lipid profile.

**Results:**

Circulating RBP4 levels were not significantly changed by bariatric surgery (4382.85±40.35 ng before, and 4393.28±33.13 ng after surgery, p=0.842), neither did adipsin (2949.68±46.86 pg before, and 2917.90±41.90 pg after surgery, p=0.535). Segregation of study participants into two age groups, 25-35 and 35-50 years of age, revealed that before surgery older age group (35-50) had higher RBP4 levels compared to younger group (25-35) (p=0.016). However, after surgery RBP4 levels were decreased in older group but not to a significant level (p=0.174). In younger age group after surgery, there was a near significant increase in RBP4 levels (p=0.052). There were no significant changes in RBP4 levels in both age groups after surgery (p=0.461). For adipsin, there were no significant differences before and after surgery in both age groups. Insulin, BMI and HOMA-IR index were decreased after surgery, however there was no correlation with RBP4 and adipsin levels.

**Conclusions:**

The present study findings do not suggest a role for RBP4 and adipsin in the improvement of insulin sensitivity in Saudi male obese population after gastric sleeve surgery. However, a decrease in RBP4 levels in older individuals after surgery needs further investigations to understand its effect on weight and glycemic control.

## INTRODUCTION

Obesity is a major risk factor of non-communicable diseases such as metabolic syndrome and diabetes mellitus.^1^,^2^ Bariatric surgeries have been introduced as the most functioning long-term management of severely obese individuals compared with diet and exercise. The concept of bariatric surgery is to minimize the overeating behaviour with maintenance of standard diet. Decreased gut size, minimized calorie intake and hormonal alterations are important mechanisms responsible for health improvement after bariatric surgery.^3^

Plasma retinol binding protein 4 (RBP4) is a protein that belongs to the lipocalin family and acts as a transporter protein for retinol (vitamin A), expressed in liver followed by visceral adipose tissue.^4^ The expression of RBP4 is higher in obese individuals compared to lean controls which is linked to increased visceral adipose tissue content.^5^ RPB4 levels were reduced after gastric banding surgery and associated with weight loss, lipid parameters, reduced waist-hip ratio and visceral fat.^6^,^7^

Adipsin is a member of serine protease family, an adipokine and identified as complement factor D.^8^ Human adipsin is mainly produced in adipose tissue, initiates triglyceride storage and inhibits lipolysis.^9^ Bariatric surgery improves adipsin levels among obese population.^10^,^11^ Hence, hormones and other relevant molecules affecting appetite, satiety, energy expenditure, and glucose metabolism are also contributory mechanisms in bariatric surgery. Therefore, this study was designed to evaluate the impact of gastric sleeve surgery on RBP4 and adipsin levels among Saudi male obese population.

## METHODS

This study was conducted in the Departments of Physiology and Surgery, College of Medicine and Obesity Research Centre (ORC), King Khalid University Hospital, King Saud University. In this study, 33 obese male patients age ranged from 25 to 50 years with obesity grade II & III, undergoing gastric sleeve surgery were enrolled. A written consent was signed by all the participants. This study was approved by Institutional Review Board, Ethics Committee, College of Medicine Research Centre, King Saud University, Riyadh, Saudi Arabia (Project number, E-17-2652, Ref: 17/0969/IRB, Dated: 05-12-2017).

### Clinical Examinations and Anthropometric measurements

Before surgical procedures, all subjects were interviewed and clinically examined by a physician, a psychologist and a nutritionist. The patients were not receiving any medicine or having any kidneys, thyroid, or liver disorders. At each visit, detailed history and BMI were recorded and patients were classified according to their BMI results.

### Biochemical Analysis

Blood samples were collected from patients in the fasting state on day of surgery and 6-12 months later and processed within 30 minutes. Plasma was separated and stored at -80°C in aliquots, until required. Routine CBC, lipid profile, glucose, insulin levels results were retrieved from the hospital files.

Plasma levels of RBP4 and adipsin were analysed by enzyme linked immunosorbent assay (ELISA) by using indirect Simple Step Human ELISA Kits (RBP4, Ab108897 and adipsin Ab99969) following the manufactures instructions, (abcam, Cambridge, UK). Assay standards and patient samples (33 pre- and 33 post-bariatric surgery) were added to respective wells of microplates and incubated at room temperature for one hour on a plate shaker. Antibody cocktails (capture and detector antibodies) were added and incubated as above. Microplates were washed three times with 1x wash buffer by an automated microplate washer (ELx50, BioTek Instruments, USA). TMB substrate 100µL was added and incubated at room temperature on plate shaker for 30 minutes. The reactions were stopped by adding 100μl stop solution and absorbance was read at 450nm by microplate reader (EL 800, BioTek Instruments, USA).

### Statistical Analysis

Data was analysed using SPSS (IBM Corp. Released 2012. IBM SPSS statistics for Windows, Version 21.0. Armonk, NY: IBM Corp.). Categorical data were expressed as absolute numbers and percentages. Numerical data were expressed as mean, median, standard error of mean (SEM) and range. Student’s t-test was used in both distributions the data pre & post-surgery, p value < 0.05 was considered statistically significant.

## RESULTS

### Demographic and biochemical assessments

The mean age and weight of the 33 obese male subjects were 35.12±0249 and 150.16±0.77 respectively. There was significant decrease in BMI after bariatric surgery (from 52.18±0.299 before surgery to 40.11±0.270 Kg/m^2^ [mean±SEM], 6-12 months after surgery, p<0.001). Levels of insulin were decreased from 19.35±0.304 mIU/L to 8.80±0.181 mIU/L, (p<0.001) and HOMA-IR index was reduced from 6.48+0.164 to 2.52±0.061 (p<0.001), as described previously.^12^

### Measurement of RBP4 and adipsin levels before and after gastric sleeve surgery

The circulating concentrations of RBP4 were not changed (4382.85±40.35 before surgery, 4393.28±33.13 ng/mL after surgery). There was no significant change in systemic adipsin levels before (2949.68±46.86) and after surgery (2917.90±41.90) (Table-I). There was no correlation between RBP4 and adipsin levels with insulin and lipid parameters (data not shown).

**Table-I:** Analysis of RBP4 and Adipsin expression pre- and post-gastric sleeve surgery.

Markers	Pre-Surgery Mean±SEM	Post-Surgery Mean±SEM	p value
RBP4 (ng/mL)	4382.85±40.35	4393.28±33.13	0.84
Adipsin (ρg/mL)	2949.68±46.86	2917.90±41.90	0.53

### Analysis of effect of age on RBP4 and adipsin levels before and after surgery

The study subjects were divided into two age groups, 25-35 and 35-50 years of age. Before surgery, older age group (35-50) had higher RBP4 levels compared to younger group (25-35) (p=0.016). However, after surgery RBP4 levels were deceased in older group but not to a significant level (p=0.174) (Table-II). In younger age group after surgery, there was a significant increase in RBP4 levels (p=0.052). However, after surgery there was no significant change in both groups (0.461) (Table-II). For adipsin, there were no differences in its levels before and after surgery in both age groups (Table-III).

**Table-II:** Effect of Age on RBP4 expression pre- and post-gastric sleeve surgery.

Age Group Years	Pre-Surgery Mean±SEM	Post-Surgery Mean±SEM	p value
25-35	4309.69±46.12	4396.11±23.68	0.052
35-50	4482.14±64.30	4389.43±72.90	0.174
p value	0.016	0.461	

**Table-III:** Effect of Age on Adipsin expression pre- and post-gastric sleeve surgery.

Age Group Years	Pre-Surgery Mean±SEM	Post-Surgery Mean±SEM	p value
25-35	2930.42±64.56	2949.04±61.05	0.779
35-50	2975.81±69.41	2875.62±54.27	0.224
p value	0.319	0.197	

## DISCUSSION

Bariatric surgical procedures are effective options for morbidly obese patients to obtain weight loss and insulin sensitivity. In this study, we investigated the impact of gastric sleeve surgery on RBP4 and adipsin levels among Saudi male obese population.

**Fig.1:**
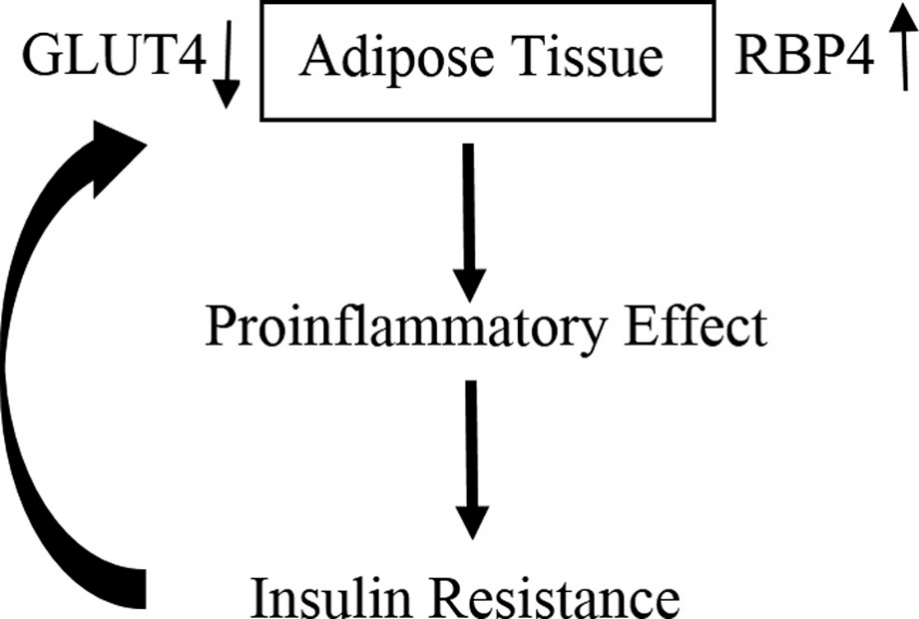
Increased secretion of RBP4 from adipose tissue has proinflammatory effect and causes insulin resistance which in turn downregulates GLUT4 and raise RBP4 levels.^25^,^26^

RBP4 is usually associated with obesity, type 2 diabetes mellitus and insulin resistance in most studies (Fig.1). We had previously shown that gene expression of RBP4 in the liver of calorically restricted mice was 13-fold higher than ad libitum fed animals, implicating a relationship with either reduced calories or body weight.^13^ In this study, circulating levels of RBP4 and adipsin were not changed after surgery. However, when the study subjects were segregated into two age groups (25-35 and 35-50 years of age), RBP4 concentration were decreased in older individuals but not to a significant level. It would be interesting to replicate this study in a larger sample size to understand the effect of gastric sleeve surgery on RBP4 levels. A decrease in RBP4 concentrations after gastric sleeve surgery in older individuals is promising as it might help to increase insulin sensitivity.

In humans, inconsistent findings have been reported for systemic concentrations of RBP4 after bariatric surgery or other weight loss interventions. Some studies reported no change after weight loss while others reported reduction in RBP4 levels. Systemic RBP4 levels are related to insulin sensitivity, lipid profiles and adiposity measurements. Both gender and age also affect RBP4 secretions.^14^,^15^ A previous study had reported higher concentrations of RBP4 in overweight women than normal weight woman and were related to weight status, while no statistical differences were noted in men.^16^ In another study, in obese women after gastric bypass surgery, RBP4 was related to weight and lipid parameters but not to insulin sensitivity.^7^ In our study, we did not find a correlation to weight, BMI and insulin levels in both old and young groups of study participants.

Elevated levels of RBP4 are also associated with higher risk of diabetes mellitus in women but not in men after adjusting risk factors, although men had higher levels of RBP4 than women.^17^ Higher expression of RBP4 mRNA in subcutaneous adipose tissue in women has been reported compared to men, however, there was no gender difference in circulating RBP4 levels.^18^ A recent study has reported no role of gender disparity in decreased RBP4 levels in Chinese obese male and female patients at three and six month intervals after laparoscopic sleeve gastrectomy. Although gender-based differences were present in glucose and lipid metabolism parameters depending upon time after surgery.^19^ Thus, various factors such as race, gender, study population and detection methods regulate RBP4 levels. Our study samples consisted of only male patients, therefore it would be interesting to repeat this study in a larger sample size including female subjects to understand the role RBP4 in this population.

**Fig.2:**
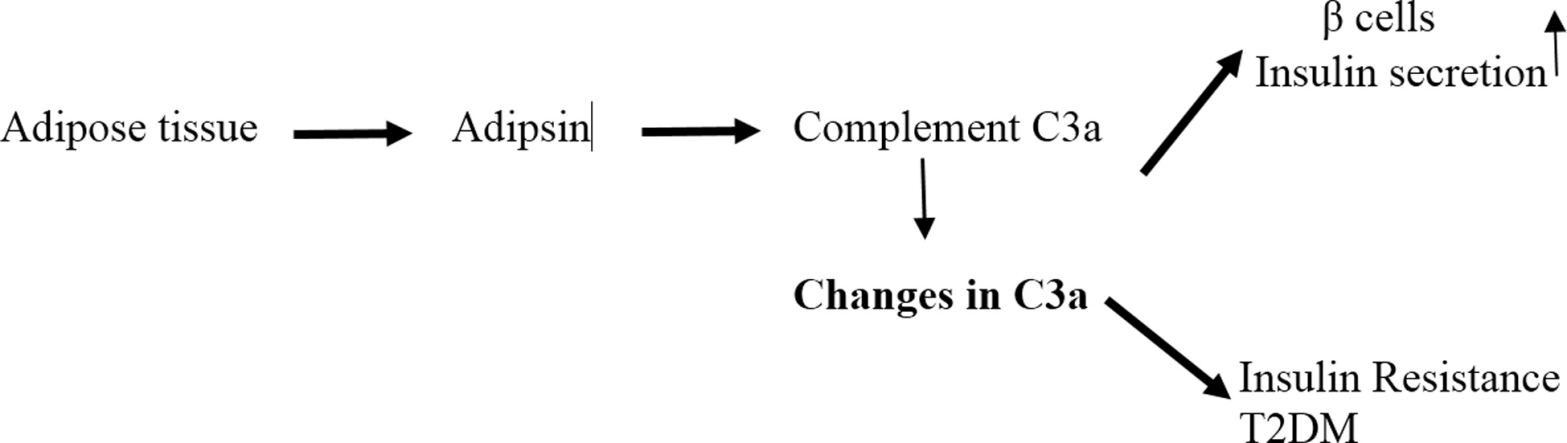
Adipsin secreted by adipose tissue generates complement C3a which stimulates insulin secretion from β cells. Changes in C3a complement lead to insulin resistance.^22^

Adipsin is one of the major protein of adipose tissue and has been shown to regulate energy homeostasis and its levels are dysregulated in obesity and diabetes (Fig.2).^20^ In our study, adipsin levels were not changed in young and old study participants before and after surgery.

Adipsin is correlated with body weight and was reported to be low in underweight starving malnourished individuals.^21^ Its levels are higher in people with normal glucose tolerance and negatively correlate with insulin resistance especially in obese people.^22^ In obesity, serum adipsin levels are higher and positively correlate with BMI and TG.^23^ In mild to moderate obese individuals, there is no decrease in circulating adipsin levels.^24^ In our study, we did not find any significant change in systemic adipsin levels after bariatric surgery and there was no correlation between adipsin and weight, BMI and insulin levels. The variations observed in these findings reflect different race, inclusion criteria and detection methods. It is important to mention that our study participant were healthy obese male subjects and this could be one of the reasons for unaltered adipsin levels after surgery.

### Study strengths and Limitations

To the best of our knowledge, this is the first study to investigate the role of RBP4 and adipsin in Saudi obese male patients after gastric sleeve surgery. This study has few limitations e.g., small sample size, only male subjects and lack of control group.

### Recommendations

Future studies should include larger sample size including female patients and control lean subjects undergoing non-bariatric surgery and in diabetic patients. Different follow-up time intervals after gastric sleeve surgery should also be investigated to understand the role of these biomarkers.

## CONCLUSIONS

The present study findings do not suggest a role for RBP4 and adipsin in the improvement of insulin sensitivity in Saudi male obese population after gastric sleeve surgery. However, a decrease in RBP4 levels in older individuals after surgery needs further investigations to understand its effect on weight and glycemic control.

### Author’s Contribution:

**SA, KA, AA, MI:** Conceived the idea, designed the proposal, statistical analysis, writing and editing of manuscript.

All authors are responsible for the accuracy and integrity of the work.
